# Serum neurofilament light chain level as a predictor of cognitive stage transition

**DOI:** 10.1186/s13195-021-00953-x

**Published:** 2022-01-07

**Authors:** Eun-Hye Lee, Hyuk Sung Kwon, Seong-Ho Koh, Seong Hye Choi, Jeong-Hwa Jin, Jee Hyang Jeong, Jae-Won Jang, Kyung Won Park, Eun-Joo Kim, Hee Jin Kim, Jin Yong Hong, Soo Jin Yoon, Bora Yoon, Ju-Hee Kang, Jong-Min Lee, Hyun-Hee Park, Jungsoon Ha

**Affiliations:** 1 Department of Neurology, Hanyang University Guri Hospital, Hanyang University College of Medicine, 153 Gyeongchun-ro, Guri, 11923 Republic of Korea; 2 Department of Neurology, Inha University School of Medicine, 27 Inhang-ro, Jung-gu, Incheon, Republic of Korea; 3 Department of Neurology, Ewha Womans University School of Medicine, Seoul, Republic of Korea; 4 Department of Neurology, Kangwon National University School of Medicine, Chuncheon, Republic of Korea; 5 Department of Neurology, Dong-A Medical Center, Dong-A University College of Medicine, Busan, Republic of Korea; 6 Department of Neurology, Pusan National University Hospital, Pusan National University School of Medicine and Medical Research Institute, Busan, Republic of Korea; 7 Department of Neurology, Samsung Medical Center, Sungkyunkwan University School of Medicine, Seoul, Republic of Korea; 8 Department of Neurology, Yonsei University Wonju College of Medicine, Wonju, Republic of Korea; 9 Department of Neurology, Eulji University Hospital, Eulji University School of Medicine, Daejeon, Republic of Korea; 10 Department of Neurology, Konyang University College of Medicine, Daejeon, Republic of Korea; 11 Department of Pharmacology, Inha University School of Medicine, Incheon, Republic of Korea; 12 Department of Biomedical Engineering, Hanyang University, Seoul, Republic of Korea; 13 GemVax & Kael Co., Ltd., Seongnam, Republic of Korea

**Keywords:** Neurofilament light chain, Alzheimer’s disease, Cognitive stage, Amyloid pathology, Cortical thickness

## Abstract

**Background:**

Neurofilament light chain (NFL) level has been suggested as a blood-based biomarker for neurodegeneration in dementia. However, the association between baseline NFL levels and cognitive stage transition or cortical thickness is unclear. This study aimed to investigate whether baseline NFL levels are associated with cognitive stage transition or cortical thickness in mild cognitive impairment (MCI) and cognitively unimpaired (CU) participants.

**Methods:**

This study analyzed data on participants from the independent validation cohort of the Korea Brain Aging Study for the Early Diagnosis and Prediction of Alzheimer’s disease (KBASE-V) study. Among the participants of KBASE-V study, 53 MCI and 146 CU participants who were followed up for ≥ 2 years and had data on the serum NFL levels were eligible for inclusion in this study. Participants were classified into three groups according to baseline serum NFL levels of low, middle, or high.

**Results:**

The Kaplan–Meier analysis showed association between the serum NFL tertiles and risk of cognitive stage transition in MCI (*P* = 0.002) and CU (*P* = 0.028) participants, analyzed separately. The same is true upon analysis of MCI and CU participants together (*P* < 0.001). In MCI participants, the highest serum NFL tertile and amyloid-beta positivity were independent predictors for cognitive stage transition after adjusting for covariates. For CU participants, only amyloid-beta positivity was identified to be an independent predictor.

**Conclusion:**

The study shows that higher serum NFL tertile levels correlate with increased risk of cognitive stage transition in both MCI and CU participants. Serum NFL levels were negatively correlated with the mean cortical thickness of the whole-brain and specific brain regions.

**Supplementary Information:**

The online version contains supplementary material available at 10.1186/s13195-021-00953-x.

## Background

Alzheimer’s disease (AD) is the most frequent cause of dementia. Globally, the number of individuals with dementia has been increasing [1]. The cascade of AD is believed to begin with extracellular accumulation of amyloid-beta (Aβ), which leads to intracellular formation of neurofibrillary tangles, synaptic dysfunction, neuronal loss, and cognitive decline [2, 3]. It is important to define biomarkers that best predict the progression of AD [2]. To identify individuals who are likely to progress to the clinical stage of MCI and AD at an early stage, many advances pertaining to AD biomarkers have been made. For example, brain atrophy and Aβ positivity can precede symptoms by years to decades [2]. However, these biomarkers are assessed by magnetic resonance imaging (MRI), positron emission tomography (PET) amyloid imaging, or cerebrospinal fluid (CSF) studies, which are expensive and/or invasive. In addition, it is unclear whether individuals with brain atrophy and/or Aβ positivity will develop dementia during their lifetime. Individual differences among clinicians in interpreting these biomarkers (degree of brain atrophy and Aβ positivity) are also limitations. Recently, neurofilament light chain (NFL) level has been proposed as a blood biomarker that can overcome these limitations [4].

NFL levels in the CSF are related to neuronal death and axonal degeneration [5]. Plasma NFL levels are higher in individuals with mild cognitive impairment (MCI) or Alzheimer’s disease dementia (ADD) than in cognitively unimpaired (CU) individuals [4]. Moreover, there is a significant correlation between NFL levels in the CSF and the blood [6]. Higher plasma NFL levels have also been associated with poor cognition, brain atrophy, and brain hypometabolism [4]. In familial AD, serum NFL levels were predictive of the rate of cortical thinning and cognitive decline [6]. A meta-analysis revealed that NFL levels in the blood and the CSF could not differentiate AD from disease mimics including vascular dementia, Lewy body dementia, Parkinson’s disease dementia, idiopathic normal pressure hydrocephalus, and posterior cortical atrophy [7]. Taken together, increased NFL levels may play an important role in stratifying individuals with early-stage dementia and those who are likely to show cognitive stage transition. However, the role of NFL as a blood biomarker of dementia to identify individuals who are likely to show cognitive stage transition is unclear. In addition, the association between serum NFL levels and cortical thickness of specific brain regions is unclear.

Therefore, we investigated whether serum NFL levels are associated with cognitive stage transition in CU or MCI individuals over a 3-year period. The clinical characteristics of the participants, according to the serum NFL tertile and the association between the serum NFL levels and cortical thickness of each specific brain region, were analyzed.

## Methods

### Participants

This study analyzed data on participants from the independent validation cohort of the Korean Brain Aging Study for the Early Diagnosis and Prediction of AD (KBASE-V) [8]. The KBASE-V contains a nationwide cohort, including 167 CU, 72 MCI, and 56 ADD participants from nine hospitals across South Korea from April 2015 to August 2016. The participants were between 55 and 90 years of age. Among these participants, the eligible patients for the current study were those with (1) CU or MCI, (2) more than 2 years of follow-up, and (3) serum NFL level data. In total, 146 CU and 53 MCI participants were included in this study.

All CU participants had normal (≥ 1.5 standard deviations [SDs] below the age-, sex-, and education-adjusted normative means) performance on four memory tests of the Korean version of the Consortium to Establish a Registry for Alzheimer’s Disease (CERAD; word list immediate recall, word list delayed recall, word list recognition, and constructional praxis recall) and had a global Clinical Dementia Rating (CDR) scale score of 0 [9–11]. MCI participants met the core clinical criteria for MCI due to AD established by the National Institute on Aging-Alzheimer’s Association (NIA-AA) workgroups [12] and the following criteria modified from the criteria proposed by Petersen et al. [13]: (1) CDR scale score of 0.5, (2) memory complaints by patients, caregivers, or clinicians; (3) a performance score < 1.5 SDs below the age-, education-, and sex-adjusted normative means for one or more of the four memory tests included in the CERAD, (4) the ability to perform independent activities of daily living (ADL) [14], and (5) absence of dementia. All participants were aged between 55 and 90 years and had a reliable informant who could provide the requested information to the investigators. The exclusion criteria were as follows: (1) presence of major psychiatric illness, (2) significant neurological or medical conditions or comorbidities that could affect cognitive function, (3) contraindications for MRI (e.g., pacemaker and claustrophobia), (4) illiteracy, (5) severe visual or hearing difficulty or serious communication or behavioral problems that could hinder clinical examination or brain imaging; (6) receiving an investigational drug; and (7) pregnancy or breastfeeding [8].

### Clinical assessment

All participants underwent yearly physical and neurological examinations including thorough diagnostic procedures that assessed participants’ cognition, abnormal behaviors, ADL, demographic characteristics, family history, current medications, vascular risk factors (the presence of hypertension, diabetes, dyslipidemia and smoking status), and other comorbidities using the Mini-Mental State Examination (MMSE) [9], Geriatric Depression Scale (Gdps) [15], Blessed Dementia Scale-ADL [16], CDR scale, and CERAD [8, 17]. Brain MRI and laboratory tests, including blood biochemistry assessment, lipid panel, complete blood count test, folate test, vitamin B12 test, venereal disease research laboratory test, thyroid function test, and apolipoprotein E (APOE) genotyping, were performed at baseline. Participants’ weight and height were measured while they were wearing light clothing. Participants’ body mass index (BMI) was calculated using their weight (kg) divided by the square of their height (m^2^).

### Brain MRI

All participants underwent brain MRI. A 3.0-T MRI scanner was used to capture three-dimensional (3D) T1- and T2-weighted SPACE sagittal images with 0.8-mm thickness. AD Neuroimaging Initiative phase 2 MRI protocols were used for brain MRI [8, 18]. The 3D T1-weighted MRI parameters were as follows: repetition time (TR) = 2300 ms, echo time (TE) = 2.14 ms, inversion time (TI) = 900 ms, flip angle (FA) = 9°, and voxel resolution = 0.8 × 0.8 × 0.8 mm^3^ in the Skyra and Trio Tim scanners (Siemens, Washington, DC, USA); TR = 7.32 ms, TE = 3.02 ms, TI = 400 ms, FA = 11°, and voxel resolution = 0.8 × 0.8 × 0.8 mm^3^ in the General Electric Discovery MR750 scanner (GE Healthcare, Milwaukee, WI, USA); and TR = shortest (6.8 ms), TE = shortest (3.1 ms), FA = 9°, and voxel resolution = 0.8 × 0.8 × 0.8 mm^3^ in the Achieva scanner (Philips Healthcare, Andover, MA, USA).

The measured MRI data were analyzed using CIVET pipeline version 2.1 (https://mcin.ca/technology/civet/) [19]. The intensity difference from inhomogeneity in the magnetic field was calibrated using the N3 intensity nonuniformity correction algorithm, and the corrected T1-weighted images were aligned to the Montreal Neurological Institute 152 standard space [20, 21]. The BET algorithm was adjusted to exclude non-brain tissue from the data [22]. The inner and outer surfaces of the cortex were estimated using a deformable spherical mesh and constrained Laplacian-based automated segmentation with proximities algorithm, respectively [23]. The cortical thickness values in the native space were obtained using the Euclidean distance between the linked vertices of the inner and outer surfaces [24]. The corrected T1-weighted images were segmented into the left and right sides of the hippocampus using FMRIB’s integrated registration and segmentation tool [25]. The volumes of the hippocampus were normalized for the total intracranial volume.

### Positive amyloid pathological change (Aβ biomarkers)

Amyloid pathological change was considered positive when individuals had an abnormal Aβ biomarker based on cortical amyloid PET ligand binding and/or low CSF Aβ42 levels [26]. In total, 159 (79.9%) participants underwent amyloid PET at baseline. Sixty participants underwent ^11^C-PiB PET and 99 participants underwent ^18^F-flutemetamol PET. The CSF was collected from 100 (50.3%) participants. In total, 184 (92.5%) participants underwent testing for Aβ biomarkers, of whom 49 (24.6%) were positive.

The PET methods for each tracer and CSF analysis have been previously described [8, 17]. The standard uptake value ratio (SUVR) was obtained using the pons as a reference region on ^18^F-flutemetamol PET and the cerebellar gray matter as the reference region on ^11^C-PiB PET. The Centiloid replication analysis was performed according to previous reports [27, 28]. Based on a previous study, elevated Aβ PET was defined as a cut-point of 10 Centiloid units [28, 29]. The levels of Aβ42, t-tau, and p-tau in the CSF were measured using the multiplex xMAP Luminex platform with INNO-BIA AlzBio3 kits (Fujirebio Europe, Ghent, Belgium). The method has been described in detail in a previous paper [8]. Based on a previous study, participants who underwent CSF studies were deemed to have AD pathology when the CSF Aβ42 level was ≤ 433.68 pg/mL [8].

### NFL

CSF NFL levels were measured using NF-light ELISA RUO kit according to the instructions provided by UmanDiagnostics. Serum NFL levels were estimated using SIMOA NF-light Advantage kit produced from Quanterix. The serum and CSF samples were prepared and provided by the nine medical centers participating in the validation cohort of the KBASE-V, including Inha University Hospital. Briefly, to measure the NFL levels, CSF samples were reacted with a biotinylated detection antibody for 1 h. The detected antigen was captured by the streptavidin-HRP complex, and then, the TMB substrate was added and incubated for 20 min under protection from light. The NFL signal was measured at 450 nm.

### Outcome

The main outcome was the cognitive stage transition (from CU to MCI or dementia and from MCI to dementia) during the 3-year study period. The diagnosis of MCI was based on the core clinical criteria for MCI established by the NIA-AA workgroups [12] and the criteria modified from the criteria proposed by Petersen et al. [13], as mentioned earlier [8, 17]. The diagnosis of dementia was based on the DSM-IV-TR criteria for dementia [30], and the diagnosis of probable ADD was based on the NIA-AA core clinical criteria [31].

### Statistical analysis

Patients were divided into three groups based on tertiles of serum NFL levels at baseline. Pearson’s chi-square test, one-way analysis of variance, or the Kruskal–Wallis test was used to compare the variables among each group. When statistically significant overall differences were detected, Pearson’s chi-square test with Bonferroni correction or Tukey’s post hoc comparisons was performed to examine differences among subgroups. The time to the outcome was assessed using the Kaplan–Meier method and between-group outcome was compared by the log-rank tests. We assessed the univariate and multivariate effects of covariates using Cox proportional hazards models to estimate the relative hazards of cognitive stage transition in MCI and CU participants assessed separately and then together. Adjusted variables were age, sex, and factors selected from the results of the univariate analysis with *P* < 0.05. In addition, variables regarded as potential confounders (serum NFL [tertile], age, sex, education level, baseline MMSE score, initial cognitive stage, hippocampal volume, Aβ positivity, APOE ε4 genotype, cortical thickness, BMI, and co-morbid hypertension, DM, or dyslipidemia) were adjusted as covariates in the analysis of total (MCI plus CU) participants. The participants were divided into two groups according to the presence of cognitive stage transition. Pearson’s chi-square test, Student’s *t* test, and the Mann–Whitney *U* test were used to evaluate differences between the two groups. Nonparametric correlation between the cortical thickness of specific brain regions and serum NFL levels was calculated using Spearman’s test as cortical thickness and serum NFL levels were not normally distributed (Kolmogorov–Smirnov test, *P* < 0.01). A two-tailed *p* value of < 0.05 was considered statistically significant. All statistical analyses were performed using SPSS for Windows version 21.0 (SPSS Inc., Chicago, IL, USA).

## Results

This study included 199 (146 CU and 53 MCI) participants aged 55–90 years (mean age ± SD: 69.3 ± 8.3 years). The follow-up period ranged from 23 to 49 months (mean ± SD: 33.4 ± 7.6 months). A total of 36 (18.1%) participants showed cognitive stage transition over the study period. Among the 146 CU participants, 14 (9.6%) participants progressed to MCI and two (1.4%) participants progressed to dementia. Among the 53 MCI participants, 19 (35.8%) participants progressed to dementia. Of the 184 (92.5%) participants who underwent testing for Aβ biomarkers, 50 (25.1%) were Aβ positive. APOE genotyping was performed in all cases; 43 (21.6%) participants were APOE ε4 carriers.

**Fig. 1:**
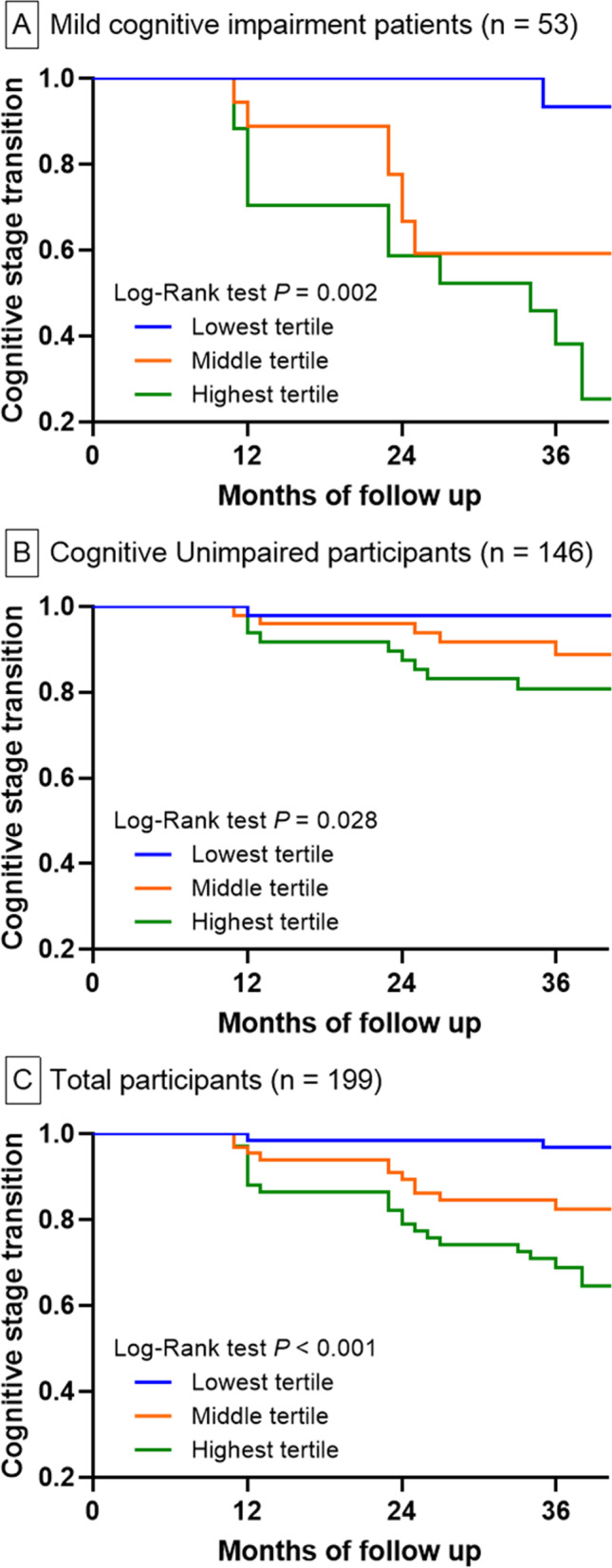
Cognitive stage transition. Kaplan–Meier curves for cognitive stage transition according to the serum NFL tertile in participants with mild cognitive impairment (**A**), cognitively unimpaired (**B**), and total participants (**C**)

The clinical characteristics of the CU and MCI participants, according to serum NFL levels, are described in Tables 1 and 2. In the MCI group, individuals with higher serum NFL tertiles were more likely to be older and showed greater tendency to have dyslipidemia, lower hippocampal volume, lower MMSE scores, and higher risk for cognitive stage transition (Table 1). In the analysis of both the CU group alone and MCI plus CU together (total participants), serum NFL tertile was found to be associated with age, education, lower MMSE score, pill intake of more than three, cortical thickness, and cognitive stage transition (Table 2 and Supplementary Table 1). There were no significant differences in sex, live alone, BMI, CDR score, Gdps score, coronary artery disease, cerebrovascular disease, cortical thickness, Aβ positivity, and APOE ε4 carrier among three groups. The Kaplan–Meier analysis showed graded associations between the serum NFL tertiles and risk of cognitive stage transition in MCI (*P* = 0.002, Fig. 1A), CU (*P* = 0.028, Fig. 1B), and total (*P* < 0.001, Fig. 1C) participants.

**Table 1:** Baseline characteristics of the MCI participants according to the initial serum NFL levels

	Lowest tertile (*n* = 18)	Middle tertile (*n* = 18)	Highest tertile (*n* = 17)	*P-*value*
Mean serum NFL, pg/ml	12.9 ± 4.0^c^	21.2 ± 3.4^b^	39.7 ± 15.5^a^	<0.001**
Range of serum NFL	5.6-18.3	18.4-23.8	24.0-84.5	
Demographics				
Age, years	67.8 ± 7.0^b^	72.8 ± 7.6^b^	79.4 ± 6.3^a^	<0.001**
Sex, female	9 (50.0)	8 (44.4)	6 (35.3)	0.676*
Lives alone	4 (22.2)	1 (5.6)	3 (17.6)	0.354*
BMI, kg/m^2^	24.0 ± 2.4	24.2 ± 2.8	24.4 ± 2.4	0.920**
Education, years	9.4 ± 3.6	9.3 ± 4.4	9.2 ± 4.3	0.993**
MMSE score, median (IQR)	25.0 (22.0 – 27.25)	24.0 (20.0 - 26.0)	21.0 (19.5-23.5)	0.041†
CDR score, median (IQR)	1.0 (1.0-1.0)	1.0 (1.0-1.0)	1.0 (1.0-1.0)	0.591†
CDR-SOB score, median (IQR)	1.0 (1.0-1.25)	1.0 (1.0-2.0)	2.0 (1.0-2.0)	0.129†
Gdps score, median (IQR)	8.5 (4.75-13.25)	10.0 (3.0-18.75)	9.0 (4.0-13.0)	0.727†
Medical history				
Hypertension	7 (38.9)	9 (50.0)	9 (52.9)	0.677*
Diabetes mellitus	4 (22.2)	2 (11.1)	5 (29.4)	0.618‡
Dyslipidemia	7 (38.9)	7 (38.9)	1 (5.9)	0.045*
Coronary artery disease	2 (11.1)	1 (5.6)	0 (0.0)	0.159‡
Cerebrovascular disease	0 (0.0)	1 (5.6)	1 (5.9)	0.362‡
Smoking	1 (5.6)	0 (0.0)	1 (5.9)	0.974‡
Pill intake of more than three	10 (55.6)	10 (55.6)	9 (52.9)	0.984*
Hippocampal volume, cm^3^	5.1 ± 0.7^a^	4.3 ± 1.4^b^	4.4 ± 0.5^ab^	0.033**
Cortical thickness, mm	3.01 ± 0.15	3.10 ± 0.17	3.07 ± 0.11	0.212**
Aβ positivity	7/17 (41.2)	11/17 (64.7)	8/15 (53.3)	0.389*
APOE ε4 carrier	3 (16.7)	6 (33.3)	3 (17.6)	0.928‡
Family history of dementia	5 (274.8)	6 (33.3)	5 (29.4)	0.933*
Cognitive stage transition	1 (5.6)^b^	7 (38.9)^ab^	11 (64.7)^a^	0.001*

Data are presented as mean ± standard deviation or number (%), unless otherwise indicated

*NFL* neurofilament light chain, *MMSE* Mini-Mental State Examination, *IQR* interquartile range, *CDR-SOB* Clinical Dementia Rating Scale Sum of Boxes, *Gdps* Geriatric Depression Scale, *Aβ* amyloid-beta

Based on *Pearson’s chi-square test, **analysis of variance, the †Kruskal–Wallis test, or ‡linear by linear association

a>b>c. Pearson’s chi-square test with Bonferroni correction or Tukey’s post hoc comparisons was performed to assess for significant differences among the subgroups

**Table 2:** Baseline characteristics of the CU participants according to the initial serum NFL levels

	Lowest tertile (*n* = 49)	Middle tertile (*n* = 49)	Highest tertile (*n* = 48)	*P-*value*
Mean serum NFL, pg/ml	11.9 ± 2.6^c^	19.1 ± 2.3^b^	37.2 ± 13.8^a^	<0.001**
Range of serum NFL, pg/ml	6.2 – 15.0	15.1 – 22.6	22.7 – 77.8	
Demographics				
Age, years	63.2 ± 6.4^b^	69.2 ± 7.3^a^	71.3 ± 7.3^a^	<0.001**
Sex, female	36 (73.5)	30 (61.2)	24 (50.0)	0.059*
Lives alone	7 (14.3)	7 (14.3)	7 (14.6)	0.999*
BMI, kg/m^2^	24.2 ± 2.6	25.0 ± 3.5	24.5 ± 2.8	0.469**
Education, years	12.9 ± 3.8^a^	10.7 ± 5.3^ab^	8.8 ± 5.3^b^	<0.001**
MMSE score, median (IQR)	28.0 (27.0 – 29.0)^a^	28.0 (25.5 - 29.0)^a^	21.0 (19.5-23.5)^b^	<0.001†
CDR score, median (IQR)	0.0 (0.0-0.0)	0.0 (0.0-0.0)	0.0 (0.0-0.0)	0.372†
CDR-SOB score, median (IQR)	0.0 (0.0-0.0)	0.0 (0.0-0.0)	0.0 (0.0-0.0)	0.045†
Gdps score, median (IQR)	7.0 (3.5-12.5)	6.0 (2.5-10.5)	8.0 (4.25-14.0)	0.241†
Medical history				
Hypertension	16 (32.7)	24 (49.0)	26 (56.5)	0.057*
Diabetes mellitus	4 (8.2)	6 (12.2)	12 (25.0)	0.054*
Dyslipidemia	18/48 (37.5)	22 (44.9)	21/46 (45.7)	0.673*
Coronary artery disease	1 (2.0)	3 (6.1)	4 (8.3)	0.174‡
Cerebrovascular disease	1 (2.0)	1 (2.0)	5 (10.4)	0.055‡
Smoking	2 (4.1)	0 (0.0)	3 (6.3)	0.565‡
Pill intake of more than three	40 (81.6)^a^	27 (55.1)^b^	24 (50.0)^b^	0.003*
Hippocampal volume, cm^3^	5.2 ± 0.9	5.0 ± 0.7	5.0 ± 0.8	0.365**
Cortical thickness, mm	3.13 ± 0.12^a^	3.10 ± 0.14^ab^	3.04 ± 0.16^b^	0.007**
Aβ positivity	6/45 (13.3)	11/45 (24.4)	7/45 (15.6)	0.345*
APOE ε4 carrier	11 (22.4)	11 (22.4)	9 (18.8)	0.877*
Family history of dementia	15 (30.6)	10 (20.4)	12 (25.0)	0.508*
Cognitive stage transition	1 (2.0)^b^	6 (12.2)^ab^	9 (18.8)^a^	0.029*

Data are presented as mean ± standard deviation or number (%), unless otherwise indicated

*NFL* neurofilament light chain, *MMSE* Mini-Mental State Examination, *IQR* interquartile range, *CDR-SOB* Clinical Dementia Rating Scale Sum of Boxes, *Gdps* Geriatric Depression Scale, *Aβ* amyloid-beta

Based on *Pearson’s chi-square test, **analysis of variance, the †Kruskal–Wallis test, or ‡linear by linear association

a>b>c. Pearson’s chi-square test with Bonferroni correction or Tukey’s post hoc comparisons was performed to assess for significant differences among the subgroups

In the univariate Cox proportional hazards regression analysis, serum NFL tertile, age, baseline MMSE score, hippocampal volume, and Aβ positivity were found to be associated with cognitive stage transition in MCI, CU, and total (MCI plus CU) participants (Table 3).

**Table 3:** Univariate Cox regression analysis of cognitive stage transition

	MCI (*n* = 53)	CU (*n* = 146)	Total (*n* = 199)
Serum NFL			
Lowest tertile	1.00 [ref]	1.00 [ref]	1.00 [ref]
Middle tertile	9.265 (1.136-75.587)*	4.968 (0.580-42.529)	6.171 (1.379–27.609)*
Highest tertile	19.569 (2.133-128.682)**	9.660 (1.224-76.272)*	11.385 (2.664–48.648)**
Age	1.118 (1.043-1.199)**	1.081 (1.013-1.154)*	1.119 (1.070–1.171)**
Sex	0.911 (0.364-2.280)	1.927 (0.698-5.316)	1.599 (0.822–3.111)
Lives alone	1.612 (0.529-4.908)	1.530 (0.432-5.425)	1.522 (0.662-3.498)
BMI	0.863 (0.711-1.047)	1.022 (0.866-1.206)	0.942 (0.831–1.068)
Education	1.016 (0.903-1.143)	0.927 (0.842-1.022)	0.951 (0.888–1.018)
Baseline MMSE score	0.818 (0.730-0.916)**	0.805 (0.702-0.923)**	0.788 (0.729–0.852)**
Initial GDS score	1.019 (0.949-1.094)	1.068 (0.997-1.144)	1.054 (1.004-1.106)*
Hypertension	1.219 (0.494-3.008)	1.007 (0.365-2.778)	1.202 (0.619-2.333)
Diabetes mellitus	1.031 (0.341-3.112)	4.099 (1.457-11.533)	2.073 (0.987-4.354)
Dyslipidemia	0.246 (0.057-1.071)	0.857 (0.305-2.408)	0.429 (0.195-0.944)*
Pill intake of more than three	0.968 (0.392-2.386)	0.662 (0.240-1.828)	0.718 (0.366-1.408)
Hippocampal volume, per 1 cm^3^	0.628 (0.447-0.882)**	0.666 (0.448-0.989)*	0.600 (0.477–0.756)**
Cortical thickness, per 1 mm	4.090 (0.226-73.875)	0.059 (0.002-1.574)	0.325 (0.032–3.288)
Aβ positivity	5.436 (1.553 – 19.030)**	10.724 (3.584-32.091)**	10.306 (4.594–23.124)**
APOE ε4 carrier	2.546 (0.981-6.661)	1.932 (0.660-5.653)	2.137 (1.057–4.3192)*
Family history of dementia	1.343 (0.526-3.430)	2.085 (0.742-5.858)	1.826 (0.914-3.648)

Data are presented as HR (95% CI)

*MCI* mild cognitive impairment, *CU* cognitive unimpaired, *NFL* neurofilament light chain, *BMI* body mass index, *MMSE* Mini-Mental State Examination, *GDS* geriatric depression scale, *HR* hazard ratio, *Aβ* amyloid-beta

*P < 0.05, **P < 0.01

The multivariate Cox proportional hazards regression analysis adjusted for covariates which include serum NFL tertiles, age, sex, baseline MMSE score, hippocampal volume, and Aβ positivity, the highest serum NFL tertile (hazard ratio [HR] 13.640, 95% confidence interval [CI] 1.346–138.270, *P* = 0.027) and Aβ positivity (HR 7.647, 95% CI 2.041–28.654, *P* = 0.003) were independent predictors of cognitive stage transition in MCI participants (Table 4). For CU participants, only Aβ positivity (HR 10.244, 95% CI 2.996–35.026, *P* < 0.001) was identified to be an independent predictor. In total (MCI plus CU) participants, the highest serum NFL tertile (HR 6.346, 95% CI 1.345–29.937, *P* = 0.020), baseline MMSE score (HR 0.866, 95% CI 0.785–0.955, *P* = 0.004), and Aβ positivity (HR8.848, 95% CI 3.614–21.662, *P* < 0.001) were independent predictor. After additional adjustment for potential confounders (education, initial cognitive stage, APOE ε4 carrier, cortical thickness, hypertension, diabetes mellitus, dyslipidemia, and BMI), NFL tertile, baseline MMSE score, and Aβ positivity remained as significant predictors (Supplementary Table 2).

**Table 4:** Multivariate Cox regression analysis of cognitive stage transition

	MCI	CU	Total
Serum NFL			
Lowest tertile	1.00 [ref]	1.00 [ref]	1.00 [ref]
Middle tertile	5.771 (0.593-56.175)	1.954 (0.196-19.535)	3.541 (0.737–17.019)
Highest tertile	13.640 (1.346-138.270)*	3.982 (0.443-35.809)	6.346 (1.345–29.937)*
Age	1.030 (0.940-1.804)	0.918 (0.921-1.095)	1.007 (0.953–1.064)
Sex	0.443 (0.109-1.804)	1.888 (0.636-5.598)	1.496 (0.694–3.226)
Baseline MMSE score	0.925 (0.799-1.070)	0.849 (0.700-1.030)	0.866 (0.785–0.955)*
Hippocampal volume, per 1 cm^3^	0.703 (0.392-1.260)	0.977 (0.499-1.911)	0.899 (0.609–1.327)
Aβ positivity	7.647 (2.041-28.654)*	10.244 (2.996-35.026)**	8.848 (3.614–21.662)**

Data are presented as hazard ratio (95% CI)

*MCI* mild cognitive impairment, *CU* cognitive unimpaired, *NFL* neurofilament light chain, *MMSE* Mini-Mental State Examination, *Aβ* amyloid-beta

*P < 0.05, **P < 0.01

Adjusted for serum NFL (tertile), age, sex, baseline MMSE score, hippocampal volume, amyloid pathology

Table 5 shows the demographic characteristics of the participants and their risk factors according to cognitive stage transition (i.e., CU to MCI or dementia and MCI to dementia) during the study period. Participants with cognitive stage transition tend to be older and were more likely to have diabetes, lower cognitive function, lower hippocampal volume, more Aβ positivity, and a higher serum NFL tertile than those without cognitive stage transition. Among participants with cognitive stage transition, 20 of 32 (62.5%) participants had Aβ positivity. Older age, lower cognitive function, lower hippocampal volume, more Aβ positivity, and a higher serum NFL tertile were noted in participants with cognitive stage transition in both the MCI and CU groups (Supplementary Tables 3 and 4).

**Table 5:** Clinical characteristics of participants based on cognitive stage transition over the study period

	Non-converter (*n* = 164)	Converter (*n* = 35)	*P*-value
Demographics			
Age, years	67.9 ± 7.8	75.9 ± 7.2	<0.001**
Female	97 (59.1)	16 (45.7)	0.145*
Lives alone	22 (13.4)	7 (20.0)	0.316*
BMI, kg/m^2^	24.5 ± 2.9	24.0 ± 2.5	0.363**
Education, years	10.6 ± 4.8	9.4 ± 5.4	0.192**
MMSE score, median (IQR)	27.0 (24.0-29.0)	23.0 (20.0-26.0)	<0.001‡
CDR score, median (IQR)	0.0 (0.0-0.0)	0.0 (0.0-0.5)	0.001‡
CDR-SOB score, median (IQR)	0.0 (0.0-0.0)	0.5 (0.0-1.0)	<0.001‡
Gdps score, median (IQR)	7.0 (4.0-11.75)	11.0 (3.0-14.0)	0.090‡
Initial cognitive stage			<0.001*
Cognitive unimpaired	130 (79.3)	49 (45.7)	
Mild cognitive impairment	34 (20.7)	19 (54.3)	
Medical history			
Hypertension	73/162 (45.1)	18 (51.4)	0.493*
Diabetes mellitus	23 (14.0)	10 (28.6)	0.036*
Dyslipidemia	68/161 (42.2)	8 (22.9)	0.033*
Coronary artery disease	9 (5.5)	2 (5.7)	1.000†
Cerebrovascular disease	6 (3.7)	3 (8.6)	0.197†
Smoking	6 (3.7)	1 (2.9)	1.000†
Pill intake of more than three	101 (61.6)	19 (54.3)	0.451*
Hippocampal volume, cm^3^	5.1 ± 0.8	4.4 ± 1.0	<0.001**
Cortical thickness, mm	3.08 ± 0.15	3.05 ± 0.14	0.289*
Positive amyloid pathology‡	27/152 (17.8)	23/32 (71.9)	<0.001*
APOE ε4 carrier	31 (18.9)	12 (34.3)	0.045*
Family history of dementia	43 (26.2)	13 (37.1)	0.393*
Initial serum NFL level, tertile			<0.001*
Lowest tertile	64 (39.0)	2 (5.7)	
Middle tertile	54 (32.9)	12 (34.3)	
Highest tertile	46 (28.0)	21 (60.0)	

*IQR* interquartile range, *NFL* neurofilament light chain

Data are presented as mean ± standard deviation or number (%). unless otherwise indicated

Based on *Pearson’s chi-square test, **Student’s t-test, the †Fisher’s exact test, or ‡Mann-Whitney *U* test

**Fig. 2:**
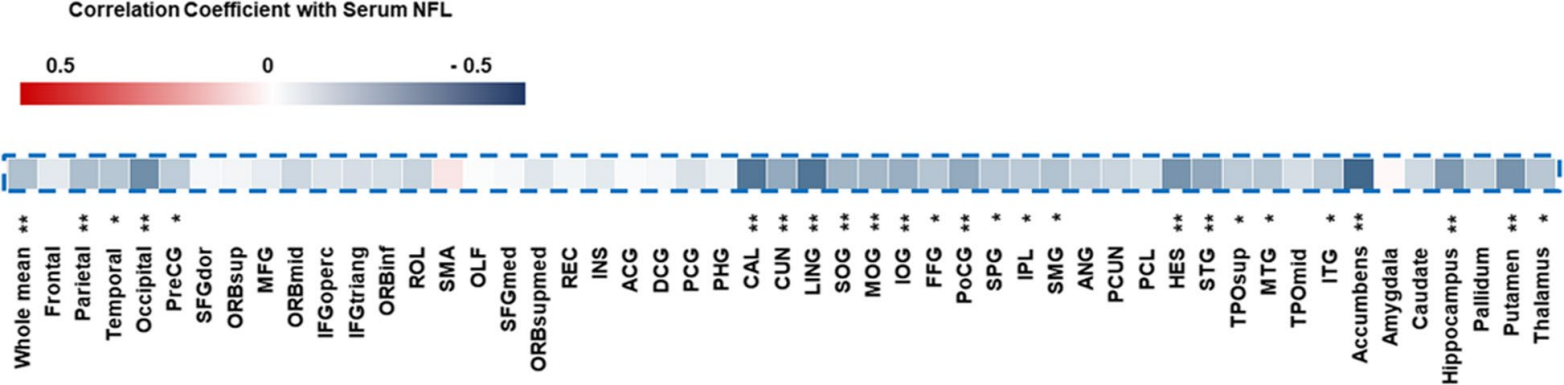
The correlation between the cortical thickness and serum NFL levels. The correlation between the cortical thickness of specific brain regions and serum NFL levels is represented using different colors. The positive and negative correlation coefficients are shown in red and blue, respectively. Statistical significance was analyzed using Spearman’s bivariate correlation (*p value < 0.05, **p value < 0.01). PreCG, precentral gyrus; SFGdor, superior frontal gyrus (dorsal); ORBsup superior orbital gyrus; MFG, middle frontal gyrus; ORBmid, middle orbital gyrus; IFGoperc, inferior frontal gyrus pars opercularis; IFGtriang, inferior frontal gyrus pars triangularis; ORBinf, inferior orbital gyrus; ROL, rolandic operculum; SMA, supplementary motor area; OLF, olfactory cortex, SFGmed, superior frontal gyrus (medial); ORBsupmed, superior frontal gyrus (medial orbital); REC, gyrus rectus; INS, insula; ACG, anterior cingulate gyrus; DCG, dorsal cingulate gyrus; PCG, posterior cingulate gyrus; PHG, parahippocampal gyrus; CAL, calcarine fissure and surrounding cortex; CUN, cuneus; LING, lingual gyrus; SOG, superior occipital gyrus; MOG, middle occipital gyrus; IOG, inferior occipital gyrus; FFG, fusiform gyrus; PoCG, postcentral gyrus; SPG, superior parietal gyrus; IPL, inferior parietal lobule; SMG, supra marginal gyrus; ANG, angular gyrus; PCUN, precuneus; PCL, paracentral lobule; HES, Heschl’s gyrus; STG, superior temporal gyrus; TPOsup, superior temporal pole; MTG, middle temporal gyrus; TPOmid, middle temporal pole; ITG, inferior temporal gyrus

Serum NFL levels were negatively correlated with the average whole-brain cortical thickness and cortical thickness of the parietal lobe, temporal lobe, and occipital lobe (Fig. 2 and Supplementary Figure 1). The cortical thickness of specific brain regions, including the hippocampus, globus pallidus, accumbens, putamen, and thalamus, were also significantly negatively correlated with serum NFL levels (Fig. 2 and Supplementary Table 5). No specific brain regions were positively correlated with serum NFL levels. Correlation between serum NFL levels and cortical thickness in MCI and CU participants, separately, are demonstrated in Supplementary Figures 2 and 3. In MCI participants, serum NFL levels were negatively correlated with the thickness of the calcarine fissure and surrounding cortex, lingual gyrus, accumbens, hippocampus, and putamen. In contrast, serum NFL levels were negatively correlated with the average whole-brain cortical thickness and cortical thickness of the parietal lobe, temporal lobe, occipital lobe, and most of the other specific brain regions in CU participants.

## Discussion

The present study demonstrated that a higher serum NFL tertile was associated with cognitive stage transition in CU and MCI participants. After adjusting for covariates, it was found that high serum NFL tertile levels and Aβ positivity were independent predictors of cognitive stage transition in MCI participants alone or MCI plus CU participants together. Serum NFL levels were negatively correlated with the cortical thickness of the whole-brain and specific brain regions, including the hippocampus, thalamus, and basal ganglia.

Progression of dementia is associated with age, baseline cognitive function [32], education level [33], hippocampal volume [34], Aβ positivity [2], and vascular risk factors [35]. However, to our knowledge, this is the first study to report that baseline serum NFL levels can help predict cognitive stage transition in total (CU and MCI) participants after adjusting for covariates, including the aforementioned factors. According to a recent study on presymptomatic familial AD, serum NFL levels were predictive of cognitive decline [6]. However, it is important to apply these findings to sporadic AD. In our study, 71.9% participants had no family history of dementia. Longitudinal plasma NFL levels have been suggested as a noninvasive biomarker for monitoring neurodegeneration in patients with AD and the effects of drugs in clinical trials [36]. Our study demonstrates that serum NFL levels may play an important role, alone or in combination with other biomarkers, to differentiate converters from non-converters.

There have been several clinical trials on AD treatments, but no treatment has been shown to modify the progression of neurodegeneration. One of the reasons for the failure these clinical trials is that they were not performed early enough to modify the disease process [37]. In addition, cognitive decline may not occur in some individuals with Aβ positivity [2]. Currently, biomarkers, including brain atrophy and Aβ positivity, are assessed using MRI, PET, or CSF studies to determine individuals who are likely to progress to ADD. However, assessment of these biomarkers is expensive and/or invasive. The use of serum NFL levels as a biomarker for neurodegenerative diseases can help overcome some of the limitations of the current biomarkers. Establishing additional biomarkers will help to identify a strong link between the combination of biomarkers and the progression of clinical symptoms in the early stage of AD.

Neurofilaments are a structural component of perikaryal, dendrites, and large myelinated axons [38, 39]. They are considered potential surrogate biomarkers of diverse neurodegenerative diseases [40]. Neurofilaments are heteropolymers requiring three subunits—light, medium, and heavy [39]. Increased NFL levels in the CSF of patients with AD are associated with neuronal death and axonal degeneration [5]. In this study, serum NFL levels were negatively correlated with the cortical thickness of the parietal, temporal, and occipital lobes, which is in line with a the finding of a previous study that showed an association between higher baseline plasma NFL levels and a decline in cortical thickness [41]. However, to our knowledge, an association between serum NFL levels and the cortical thickness of specific brain regions has not been reported previously. Regarding the volume of specific brain regions, its relationship with NFL levels differed among previous reports. In a study of genetic frontotemporal dementia, NFL levels in the CSF were negatively correlated with the volume of the frontal, parietal, temporal, insular, and cingulate cortices, but they were positively correlated with the volume of the occipital cortex [42]. Another study demonstrated that baseline and longitudinal plasma NFL levels were associated with the composite volume of the hippocampus and entorhinal and temporal cortices [36]. The reason for the different associations between serum NFL levels and specific brain regions is unclear. NFL levels may reflect the degree of neurodegeneration in any brain region [40], rather than having a deep relationship with specific brain regions.

Furthermore, in our study, highest serum NFL tertile in individuals with negative amyloid pathology were associated with cognitive stage transition (*P* = 0.012, Supplementary Table 4). None of the individuals with both negative amyloid pathology and the lowest serum NFL tertile showed cognitive stage transition. These results indicate that NFL level may be a biomarker that nonspecifically reflects neurodegeneration and is not disease-specific to AD. A previous study has also suggested that NFL level might be a general biomarker for axonal degeneration rather than a tool to differentiate AD from other types of dementia [4]. However, measuring NFL levels may help screen out individuals who are likely to show cognitive stage transition.

This study has several limitations. First, participants of a single ethnicity were included. Second, the median follow-up period was only 3 years. This short follow-up period might be insufficient to show the difference in the relationship of cognitive stage transition and serum NFL tertile in CU participants. Thus, the relationship between serum NFL levels and cognitive stage transition was found to be more prominent in the MCI group than in the CU group. Including both normal and subjective cognitive decline participants in the CU group and more participants with a family history of dementia in the MCI group than in the CU group (32.1% vs. 26.7%, respectively) might be other reasons for this difference. However, all participants in this study were followed up for more than 2 years, which could help determine the specific target in clinical trials. Third, some non-amnestic MCI patients might have been classified into the CU group due to the inclusion criteria of scoring above age-, sex-, and education-adjusted memory scales. However, these criteria are in line with Alzheimer’s disease neuroimaging initiative 3 protocol [43]. Also, all CU participants in current study showed normal cognitive function in all domains except two participants who showed performance scores that were < 1.5 SDs below the normative means for visuospatial function. Fourth, we allowed three 3D T1-weighted MRI parameters in this cohort. Although the difference from inhomogeneity in the magnetic field was calibrated using the N3 intensity nonuniformity correction algorithm, there might be remaining differences depending on scanner types that we failed to consider. Fifth, serum NFL levels were only measured at baseline. However, it would be useful to predict cognitive stage transition using serum NFL levels measured at single time point. Finally, NFL levels in the CSF were measured in only half (*n* = 100) of the participants. However, the correlation coefficient between the CSF and serum NFL levels was 0.358 (*p* < 0.001, Supplementary Figure 4).

## Conclusions

In conclusion, high serum NFL levels in MCI and CU participants indicated that they had a high risk of cognitive stage transition. Furthermore, serum NFL levels were negatively correlated with the cortical thickness of the whole-brain and specific brain regions.

## Supplementary Information

**Additional file 1: Supplementary Table 1.** Baseline characteristics of the total participants according to the initial serum NFL levels**. Supplementary Table 2.** Multivariate Cox regression analysis of the factors for cognitive stage transition in total participants**. Supplementary Table 3.** Clinical characteristics of participants based on cognitive stage transition over the study period in mild cognitive impairment participants (n = 53)**. Supplementary Table 4.** Clinical characteristics of participants based on cognitive stage transition over the study period in cognitively unimpaired participants (n = 146)**. Supplementary Table 5.** Correlation between cortical thickness and neurofilament light chain levels in the serum**.** The cortical thickness of specific brain regions, including the hippocampus, globus pallidus, accumbens, putamen, and thalamus, were significantly negatively correlated with serum NFL levels. **Supplementary Table 6.** Clinical characteristics of participants with negative amyloid pathology according to the initial serum NFL levels (n = 135)**.** Higher serum NFL levels in individuals with negative amyloid pathology were associated with cognitive stage transition (*P* = 0.003).

**Additional file 2: Supplementary Figure 1.** The correlation between the cortical thickness of each specific brain region and serum NFL. The correlation between the cortical thickness of each specific brain region and serum NFL is represented in different colors. The positive and negative correlation coefficients are shown in red and blue, respectively. The cortical thickness between each brain region showed a positive correlation. NFL levels were negatively correlated with the cortical thickness of the whole-brain and specific brain regions. PreCG, precentral gyrus; SFGdor, superior frontal gyrus (dorsal); ORBsup superior orbital gyrus; MFG, middle frontal gyrus; ORBmid, middle orbital gyrus; IFGoperc, inferior frontal gyrus pars opercularis; IFGtriang, inferior frontal gyrus pars triangularis; ORBinf, inferior orbital gyrus; ROL, rolandic operculum; SMA, supplementary motor area; OLF, olfactory cortex; SFGmed, superior frontal gyrus (medial); ORBsupmed, superior frontal gyrus (medial orbital); REC, gyrus rectus; INS, insula; ACG, anterior cingulate gyrus; DCG, dorsal cingulate gyrus; PCG, posterior cingulate gyrus, PHG, parahippocampal gyrus; CAL, calcarine fissure and surrounding cortex; CUN, cuneus; LING, lingual gyrus; SOG, superior occipital gyrus; MOG, middle occipital gyrus; IOG, inferior occipital gyrus; FFG, fusiform gyrus; PoCG, postcentral gyrus; SPG, superior parietal gyrus; IPL, inferior parietal lobule; SMG, supra marginal gyrus; ANG, angular gyrus; PCUN, precuneus, PCL, paracentral lobule; HES, Heschl’s gyrus; STG, superior temporal gyrus; TPOsup, superior temporal pole; MTG, middle temporal gyrus; TPOmid, middle temporal pole; ITG, inferior temporal gyrus.

**Additional file 3: Supplementary Figure 2.** The correlation between the cortical thickness of each specific brain region and serum NFL in mild cognitive impairment participants (n = 51). The correlation between the cortical thickness of each specific brain region and serum NFL is represented in different colors. Positive and negative correlation coefficients are shown in red and blue, respectively. NFL levels were negatively correlated with the cortical thickness of the specific brain regions including the CAL, LING, accumbens, hippocampus, and putamen. PreCG, precentral gyrus; SFGdor, superior frontal gyrus (dorsal); ORBsup superior orbital gyrus; MFG, middle frontal gyrus; ORBmid, middle orbital gyrus; IFGoperc, inferior frontal gyrus pars opercularis; IFGtriang, inferior frontal gyrus pars triangularis; ORBinf, inferior orbital gyrus; ROL, rolandic operculum; SMA, supplementary motor area; OLF, olfactory cortex; SFGmed, superior frontal gyrus (medial); ORBsupmed, superior frontal gyrus (medial orbital); REC, gyrus rectus; INS, insula; ACG, anterior cingulate gyrus; DCG, dorsal cingulate gyrus; PCG, posterior cingulate gyrus, PHG, parahippocampal gyrus; CAL, calcarine fissure and surrounding cortex; CUN, cuneus; LING, lingual gyrus; SOG, superior occipital gyrus; MOG, middle occipital gyrus; IOG, inferior occipital gyrus; FFG, fusiform gyrus; PoCG, postcentral gyrus; SPG, superior parietal gyrus; IPL, inferior parietal lobule; SMG, supra marginal gyrus; ANG, angular gyrus; PCUN, precuneus, PCL, paracentral lobule; HES, Heschl’s gyrus; STG, superior temporal gyrus; TPOsup, superior temporal pole; MTG, middle temporal gyrus; TPOmid, middle temporal pole; ITG, inferior temporal gyrus.

**Additional file 4: Supplementary Figure 3.** The correlation between the cortical thickness of each specific brain region and serum NFL in cognitively unimpaired participants (n = 145). The correlation between the cortical thickness of each specific brain region and serum NFL is represented in different colors. Positive and negative correlation coefficients are shown in red and blue, respectively. NFL levels were negatively correlated with the cortical thickness of the whole-brain and specific brain regions including the parietal, temporal, and occipital cortex. PreCG, precentral gyrus; SFGdor, superior frontal gyrus (dorsal); ORBsup superior orbital gyrus; MFG, middle frontal gyrus; ORBmid, middle orbital gyrus; IFGoperc, inferior frontal gyrus pars opercularis; IFGtriang, inferior frontal gyrus pars triangularis; ORBinf, inferior orbital gyrus; ROL, rolandic operculum; SMA, supplementary motor area; OLF, olfactory cortex; SFGmed, superior frontal gyrus (medial); ORBsupmed, superior frontal gyrus (medial orbital); REC, gyrus rectus; INS, insula; ACG, anterior cingulate gyrus; DCG, dorsal cingulate gyrus; PCG, posterior cingulate gyrus, PHG, parahippocampal gyrus; CAL, calcarine fissure and surrounding cortex; CUN, cuneus; LING, lingual gyrus; SOG, superior occipital gyrus; MOG, middle occipital gyrus; IOG, inferior occipital gyrus; FFG, fusiform gyrus; PoCG, postcentral gyrus; SPG, superior parietal gyrus; IPL, inferior parietal lobule; SMG, supra marginal gyrus; ANG, angular gyrus; PCUN, precuneus, PCL, paracentral lobule; HES, Heschl’s gyrus; STG, superior temporal gyrus; TPOsup, superior temporal pole; MTG, middle temporal gyrus; TPOmid, middle temporal pole; ITG, inferior temporal gyrus.

**Additional file 5: Supplementary Figure 4.** Serum and cerebrospinal fluid neurofilament light chain according to amyloid-beta positivity. Serum and cerebrospinal fluid neurofilament light chain according to amyloid-beta positivity. Fit lines are shown for each group. The Spearman’s ρ and P values relate to Spearman’s rank correlation for each group. NFL, neurofilament light chain; CSF, cerebrospinal fluid.

## Data Availability

The datasets used and analyzed during the current study are available from the corresponding author on reasonable request.

## Abbreviations

AD: Alzheimer’s disease
ADD: Alzheimer’s disease dementia
BMI: Body mass index
CDR: Clinical dementia rating
CU: Cognitively unimpaired
DM: Diabetes mellitus
GE: General electric
MCI: Mild cognitive impairment
MMSE: Mini-Mental State Examination
NCEP: National cholesterol education program
OR: Odds ratio
SD: Standard deviation
SUVR: Standard uptake value ratio
