# Supplementary material for: Serum neurofilament light chain level as a predictor of cognitive stage transition
Source: Alzheimers Res Ther. 2022 Jan 7;14:6. doi: 10.1186/s13195-021-00953-x (PMC8742445; doi:10.1186/s13195-021-00953-x)
Supplement: Supplementary file 1 — Additional file 1: Supplementary Table 1. Baseline characteristics of the total participants according to the initial serum NFL levels. Supplementary Table 2. Multivariate Cox regression analysis of the factors for cognitive stage transition in total participants. Supplementary Table 3. Clinical characteristics of participants based on cognitive stage transition over the study period in mild cognitive impairment participants (n = 53). Supplementary Table 4. Clinical characteristics of participants based on cognitive stage transition over the study period in cognitively unimpaired participants (n = 146). Supplementary Table 5. Correlation between cortical thickness and neurofilament light chain levels in the serum. The cortical thickness of specific brain regions, including the hippocampus, globus pallidus, accumbens, putamen, and thalamus, were significantly negatively correlated with serum NFL levels. Supplementary Table 6. Clinical characteristics of participants with negative amyloid pathology according to the initial serum NFL levels (n = 135). Higher serum NFL levels in individuals with negative amyloid pathology were associated with cognitive stage transition (P = 0.003). [file 13195_2021_953_MOESM1_ESM.doc]

**Supplementary Table 1.** Baseline characteristics of the total participants according to the initial serum NFL levels

|  | Lowest tertile  (n = 66) | Middle tertile  (n = 66) | Highest tertile  (n = 67) | *P-*value* |
| --- | --- | --- | --- | --- |
| Demographics |  |  |  |  |
| Age, years | 64.3 ± 6.7^c^ | 69.9 ± 7.5^b^ | 73.6 ± 7.9^a^ | <0.001 |
| Sex, female | 45 (68.2) | 36 (54.5) | 32 (47.8) | 0.054 |
| Lives alone | 9 (13.6) | 10 (15.2) | 10 (15.4) | 0.954 |
| BMI, kg/m^2^ | 24.2 ± 2.5 | 24.8 ± 3.3 | 24.4 ± 2.7 | 0.462 |
| Education, years | 11.9 ± 4.0^a^ | 10.5 ± 5.2^ab^ | 8.8 ± 5.0^b^ | 0.001 |
| MMSE score, median (IQR) | 28.0 (26.0 - 29.0) | 26.5 (22.75 - 29.0) | 24.0 (22.0 - 27.0) | < 0.001 |
| CDR score, median (IQR) | 0.0 (0.0-0.0) | 0.0 (0.0-0.5) | 0.0 (0.0-0.5) | 0.539 |
| CDR-SOB score, median (IQR) | 0.0 (0.0-0.0) | 0.0 (0.0-0.5) | 0.0 (0.0-0.5) | 0.127 |
| Gdps score, median (IQR) | 7.0 (4.0-12.0) | 7.5 (3.0-11.5) | 8.0 (4.0-13.0) | 0.606 |
| Initial cognitive stage |  |  |  | 0.471 |
| Cognitive unimpaired | 52 (78.8) | 47 (71.2) | 47 (70.1) |  |
| Mild cognitive impairment | 14 (21.2) | 19 (28.8) | 20 (29.9) |  |
| Medical history |  |  |  |  |
| Hypertension | 21 (31.8)^b^ | 33 (50.0)^ab^ | 37/65 (56.9)^a^ | 0.012 |
| Diabetes mellitus | 6 (9.1) | 10 (15.2) | 17 (25.4) | 0.038 |
| Dyslipidemia | 25/65 (38.5) | 28 (42.4) | 23/65 (35.4) | 0.709 |
| Coronary artery disease | 3 (4.5) | 4 (6.1) | 4 (6.0) | 0.913 |
| Cerebrovascular disease | 1 (1.5) | 2 (3.0) | 6 (9.0) | 0.092 |
| Smoking | 3 (4.5) | 0 (0.0) | 4 (6.0) | 0.150 |
| Pill intake of more than three | 48 (72.7)^b^ | 38 (57.6)^ab^ | 34 (52.3)^a^ | 0.045 |
| Hippocampal volume, cm^3^ | 5.2 ± 0.9^a^ | 4.8 ± 1.0^b^ | 4.8 ± 1.0^b^ | 0.009 |
| Cortical thickness, mm | 3.11 ± 0.13 | 3.09 ± 0.16 | 3.05 ± 0.18 | 0.072 |
| Aβ positivity | 15/61 (24.6) | 19/62 (30.6) | 16/61 (26.2) | 0.737 |
| APOE ε4 carrier | 13 (19.7) | 18 (27.3) | 12 (17.9) | 0.380 |
| Initial serum NFL, pg/ml | 12.0 ± 2.7^c^ | 19.5 ± 2.1^b^ | 37.6 ± 14.1^a^ | <0.001 |
| Family history of dementia | 21 (31.8) | 15 (22.7) | 20 (29.9) | 0.473 |
| Cognitive stage transition | 2 (3.0)^b^ | 12 (18.2)^a^ | 21 (31.3)^a^ | <0.001 |

Data are presented as mean ± standard deviation or number (%), unless otherwise indicated.

NFL, neurofilament light chain; MMSE, Mini-Mental State Examination; IQR, interquartile range; CDR-SOB, Clinical Rating Scale sum of boxes; Gdps, Geriatric Depression Scale; Aβ, amyloid-beta

*Based on Pearson’s chi-square test, analysis of covariance, or the Kruskal–Wallis test.

a>b>c. Pearson’s chi-square test with Bonferroni correction or Tukey’s post hoc comparisons was performed to assess for significant differences among the subgroups.

Supplementary Table 2. Multivariate Cox regression analysis of the factors for cognitive stage transition in total participants

|  | Adjusted HR model 1 | *P*-value | Adjusted HR model 2 | *P*-value |
| --- | --- | --- | --- | --- |
| Serum NFL |  |  |  |  |
| Lowest tertile | 1.00 |  | 1.00 |  |
| Middle tertile | 3.302 (0.656–16.616) | 0.147 | 3.597 (0.662–19.552) | 0.138 |
| Highest tertile | 6.494 (1.317–32.024) | 0.022 | 8.144 (1.592–41.655) | 0.012 |
| Age | 1.011 (0.954–1.072) | 0.708 | 1.009 (0.939–1.084) | 0.806 |
| Sex | 1.542 (0.618–3.848) | 0.353 | 1.496 (0.595–3.762) | 0.392 |
| Education | 1.017 (0.919–1.125) | 0.749 | 1.034 (0.931–1.148) | 0.530 |
| BMI | 0.965 (0.835–1.116) | 0.635 | 0.991 (0.862–1.139) | 0.896 |
| Baseline MMSE score | 0.858 (0.769–0.957) | 0.006 | 0.874 (0.784–0.975) | 0.016 |
| Initial cognitive stage, MCI | 0.873 (0.360-2.119) | 0.764 | 0.612 (0.237–1.579) | 0.310 |
| Hippocampal volume, per 0.1 cm^3^ | 0.990 (0.949–1.032) | 0.625 | 0.988 (0.945–1.033) | 0.606 |
| Aβ positivity | 8.297 (3.123–22.042) | <0.001 | 10.909 (3.805–31.279) | <0.001 |
| APOE ε4 carrier | 0.908 (0.401–2.252) | 0.908 | 0.762 (0.294–1.975) | 0.576 |
| Cortical thickness, per 1 mm | 1.512 (0.133–17.247) | 0.739 | 1.518 (0.123–18.799) | 0.745 |

*p* for multivariate models.

HR, hazard ratio; NFL, neurofilament light chain; BMI, body mass index; MMSE, Mini-Mental State Examination; Aβ, amyloid-beta

Data are presented as odds ratios (95% confidence intervals)

Model 1: Adjusted for serum NFL (tertile), age, sex, education, baseline MMSE score, initial cognitive stage, hippocampal volume, Aβ positivity, APOE ε4 carrier, and cortical thickness.

Model 2: Additionally, adjusted for hypertension, diabetes mellitus, dyslipidemia, and BMI.

**Supplementary Table 3.** Clinical characteristics of participants based on cognitive stage transition over the study period in mild cognitive impairment participants (n = 53)

|  | Non-converter  (n = 34) | Converter  (n = 19) | *P*-value* |
| --- | --- | --- | --- |
| Demographics |  |  |  |
| Age, years | 70.4 ± 7.5 | 78.4 ± 7.4 | <0.001* |
| Female | 15 (44.1) | 8 (42.1) | 0.887 |
| Lives alone | 4 (11.8) | 4 (21.1) | 0.436** |
| BMI, kg/m^2^ | 24.6 ± 2.7 | 23.5 ± 2.0 | 0.119* |
| Education, years | 9.2 ± 3.6 | 9.5 ± 4.7 | 0.764* |
| MMSE score, median (IQR) | 24.0 (22.0-27.0) | 21.0 (19.0-24.0) | 0.007 |
| CDR score, median (IQR) | 1.0 (1.0-1.0) | 1.0 (1.0-1.0) | 0.056 |
| CDR-SOB score, median (IQR) | 1.0 (1.0-2.0) | 1.0 (1.0-2.0) | 0.231 |
| Gdps score, median (IQR) | 8.5 (4.75-15.0) | 10.0 (3.0-13.0) | 0.816 |
| Medical history |  |  |  |
| Hypertension | 15 (44.1) | 10 (52.6) | 0.552 |
| Diabetes mellitus | 7 (20.6) | 4 (21.1) | 0.968 |
| Hyperlipidemia | 13 (38.2) | 2 (10.5) | 0.055** |
| Coronary artery disease | 3 (8.8) | 0 (0.0) | 0.545** |
| Cerebrovascular disease | 1 (2.9) | 1 (5.3) | 1.000** |
| Smoking | 1 (2.9) | 1 (5.3) | 1.000** |
| Pill intake of more than three | 19 (55.9) | 10 (52.6) | 0.820 |
| Hippocampal volume, cm^3^ | 4.9 ± 0.8 | 4.1 ± 1.1 | 0.002* |
| Cortical thickness, mm | 3.05 ± 0.15 | 3.08 ± 0.13 | 0.466* |
| Aβ positivity ‡ | 12/32 (37.5) | 14/17 (82.4) | 0.003 |
| APOE ε4 carrier | 5 (14.7) | 7 (36.8) | 0.065 |
| Family history of dementia | 9 (26.5) | 7 (36.8) | 0.430 |
| Initial serum NFL level, tertile |  |  | 0.001 |
| Lowest tertile | 16 (47.1) | 1 (5.3) |  |
| Middle tertile | 12 (35.3) | 6 (31.6) |  |
| Highest tertile | 6 (17.6) | 12 (63.2) |  |

IQR, interquartile range; NFL, neurofilament light chain; Aβ, amyloid-beta

Data are presented as mean ± standard deviation or number (%), unless otherwise indicated.

*P*-value is based on Pearson’s chi-square test, * Student’s t-test, **Fisher’s exact test, or †Mann–Whitney *U* test.

**Supplementary Table 4.** Clinical characteristics of participants based on cognitive stage transition over the study period in cognitively unimpaired participants (n = 146)

|  | Non-converter  (n = 130) | Converter  (n = 16) | *P*-value* |
| --- | --- | --- | --- |
| Demographics |  |  |  |
| Age, years | 67.3 ± 7.8 | 72.9 ± 5.9 | 0.006* |
| Female | 82 (63.1) | 8 (50.0) | 0.310 |
| Lives alone | 18 (13.8) | 3 (18.8) | 0.598 |
| BMI, kg/m^2^ | 24.5 ± 3.0 | 24.7 ± 2.9 | 0.801* |
| Education, years | 9.3 ± 6.3 | 11.0 ± 4.9 | 0.214* |
| MMSE score, median (IQR) | 28.0 (26.0-29.0) | 24.5 (22.0-26.75) | 0.001† |
| CDR score, median (IQR) | 0.0 (0.0-0.0) | 0.0 (0.0-0.0) | 0.004† |
| CDR-SOB score, median (IQR) | 0.0 (0.0-0.0) | 0.0 (0.0-0.0) | 0.212† |
| Gdps score, median (IQR) | 7.0 (3.75-10.25) | 12.0 (4.25-15.5) | 0.071† |
| Medical history |  |  |  |
| Hypertension | 58/128 (45.3) | 8(50.0) | 0.723 |
| Diabetes mellitus | 16 (12.3) | 6 (37.5) | 0.008 |
| Hyperlipidemia | 55/127 (43.3) | 6 (37.5) | 0.656 |
| Coronary artery disease | 6 (4.6) | 2 (12.5) | 0.213** |
| Cerebrovascular disease | 5 (3.8) | 2 (12.5) | 0.171** |
| Smoking | 5 (3.8) | 0 (0.0) | 1.000** |
| Pill intake of more than three | 82 (63.1) | 9 (56.3) | 0.595 |
| Hippocampal volume, cm^3^ | 5.1 ± 0.8 | 4.7 ± 1.0 | 0.041* |
| Cortical thickness, mm | 3.09 ± 0.14 | 3.04 ± 0.15 | 0.111* |
| Positive amyloid pathology‡ | 15/120 (12.5) | 9/15 (60.0) | <0.001 |
| APOE ε4 carrier | 26 (20.0) | 5 (31.3) | 0.299 |
| Family history of dementia | 31 (23.8) | 6 (37.5) | 0.236 |
| Initial serum NFL level, tertile |  |  | 0.029 |
| Lowest tertile | 48 (36.9) | 1 (6.3) |  |
| Middle tertile | 43 (33.1) | 6 (37.5) |  |
| Highest tertile | 39 (30.0) | 9 (56.2) |  |

IQR, interquartile range; NFL, neurofilament light chain

Data are presented as mean ± standard deviation or number (%). unless otherwise indicated.

*P*-value is based on Pearson’s chi-square test, * Student’s t-test, **Fisher’s exact test, or †Mann–Whitney *U* test.

‡Measured in 120 cases in the non-converter group and 15 in the converter group.

**Supplementary Table 5**. Correlation between cortical thickness and neurofilament light chain levels in the serum

|  | NFL serum correlation coefficient (n=196) | *P*-value* |
| --- | --- | --- |
| Total_intracranial_volume | 0.77 | 0.284 |
| Whole_mean | -0.175 | 0.014* |
| Frontal | -0.060 | 0.407 |
| Parietal | -0.181 | 0.011* |
| Temporal | -0.158 | 0.027* |
| Occipital | -0.316 | 0.000* |
| PreCG | -0.143 | 0.045* |
| SFGdor | -0.021 | 0.769 |
| ORBsup | -0.026 | 0.714 |
| MFG | -0.051 | 0.475 |
| ORBmid | -0.110 | 0.124 |
| IFGoperc | -0.077 | 0.285 |
| IFGtriang | -0.091 | 0.206 |
| ORBinf | -0.090 | 0.212 |
| ROL | -0.119 | 0.097 |
| SMA | 0.050 | 0.488 |
| OLF | -0.008 | 0.915 |
| SFGmed | -0.017 | 0.808 |
| ORBsupmed | -0.068 | 0.345 |
| REC | -0.028 | 0.700 |
| INS | -0.059 | 0.410 |
| ACG | -0.011 | 0.875 |
| DCG | -0.021 | 0.773 |
| PCG | -0.084 | 0.244 |
| PHG | -0.039 | 0.584 |
| CAL | -0.382 | 0.000* |
| CUN | -0.237 | 0.001* |
| LING | -0.386 | 0.000* |
| SOG | -0.204 | 0.004* |
| MOG | -0.200 | 0.005* |
| IOG | -0.224 | 0.002* |
| FFG | -0.162 | 0.023* |
| PoCG | -0.240 | 0.001* |
| SPG | -0.162 | 0.023* |
| IPL | -0.148 | 0.039* |
| SMG | -0.171 | 0.016* |
| ANG | -0.131 | 0.067 |
| PCUN | -0.118 | 0.100 |
| PCL | -0.090 | 0.209 |
| HES | -0.299 | 0.000* |
| STG | -0.242 | 0.001* |
| TPOsup | -0.144 | 0.043* |
| MTG | -0.160 | 0.025* |
| TPOmid | -0.090 | 0.208 |
| ITG | -0.152 | 0.034* |
| Accumbens | -0.421 | 0.000* |
| Amygdala | 0.016 | 0.828 |
| Caudate | -0.104 | 0.149 |
| Hippocampus | -0.286 | 0.000* |
| Pallidum | -0.137 | 0.055 |
| Putamen | -0.301 | 0.000* |
| Thalamus | -0.156 | 0.029* |

P-value (* p < 0.05) based on Spearman’s bivariate correlation analysis.

NLF, neurofilament light chain

The cortical thickness was analyzed using the mean of both sides.

PreCG, precentral gyrus; SFGdor, superior frontal gyrus (dorsal); ORBsup, superior orbital gyrus, MFG, middle frontal gyrus; ORBmid, middle orbital gyrus; IFGoperc, inferior frontal gyrus pars opercularis; IFGtriang, inferior frontal gyrus pars triangularis; ORBinf, inferior orbital gyrus; ROL, rolandic operculum; SMA, supplementary motor area; OLF, olfactory cortex; SFGmed, superior frontal gyrus (medial); ORBsupmed, superior frontal gyrus (medial orbital); REC, gyrus rectus; INS, insula; ACG, anterior cingulate gyrus; DCG, dorsal cingulate gyrus; PCG, posterior cingulate gyrus; PHG, parahippocampal gyrus; CAL, calcarine fissure and surrounding cortex; CUN, cuneus; LING, lingual gyrus; SOG, superior occipital gyrus; MOG, middle occipital gyrus; IOG, inferior occipital gyrus; FFG, fusiform gyrus; PoCG, postcentral gyrus; SPG, superior parietal gyrus; IPL, inferior parietal lobule; SMG, supra marginal gyrus; ANG, angular gyrus; PCUN, precuneus; PCL, paracentral lobule; HES, Heschl’s gyrus; STG, superior temporal gyrus; TPOsup, superior temporal pole; MTG, middle temporal gyrus; TPOmid, middle temporal pole; ITG, inferior temporal gyrus.

**Supplementary Table 6.** Clinical characteristics of participants with negative amyloid pathology according to the initial serum NFL levels (n = 135)

|  | Lowest tertile  (n = 44) | Middle tertile  (n = 45) | Highest tertile  (n = 45) | *P-*value* |
| --- | --- | --- | --- | --- |
| Demographics |  |  |  |  |
| Age, years | 62.7 ± 5.6^c^ | 69.3 ± 7.3^b^ | 71.4 ± 7.1^a^ | <0.001 |
| Sex, female | 32 (72.4)^a^ | 26 (57.8)^ab^ | 21 (46.7)^b^ | 0.043 |
| Education, years | 12.3 ± 4.2^a^ | 10.6 ± 5.3^ab^ | 8.6 ± 5.1^b^ | 0.002 |
| MMSE score, median (IQR) | 28.0 (27.0 - 29.0)^a^ | 27.0 (24.0 - 29.0)^a^ | 24.0 (22.0 - 27.0)^b^ | <0.001 |
| CDR score, median (IQR) | 0.0 (0.0-0.0) | 0.0 (0.0-0.0) | 0.0 (0.0-0.0) | 0.833 |
| CDR-SOB score, median (IQR) | 0.0 (0.0-0.0) | 0.0 (0.0-0.0) | 0.0 (0.0-0.0) | 0.579 |
| Gdps score, median (IQR) | 7.0 (3.25-11.775) | 6.0 (3.5-10.0) | 8.0 (4.5-12.5) | 0.533 |
| Hippocampal volume, cm^3^ | 5.3 ± 1.0 | 5.0 ± 0.7 | 4.9 ± 0.8 | 0.259 |
| Cortical thickness, mm | 3.11 ± 0.12 | 3.10 ± 0.14 | 3.05 ± 0.15 | 0.056 |
| APOE ε4 carrier | 8 (18.2) | 5 (11.1) | 7 (15.6) | 0.639 |
| Progression of disease | 0 (0.0)^b^ | 3 (6.7)^ab^ | 6 (13.3)^a^ | 0.012 |

Data are presented as the mean ± standard or number (%), unless otherwise indicated.

NFL, neurofilament light chain; MMSE, Mini-Mental State Examination; IQR, interquartile range; CDR-SOB, clinical rating scale sum of boxes; Gdps, geriatric depression scale

*Based on Pearson’s chi-square test, analysis of covariance, Kruskal–Wallis test, or the linear by linear association analysis

a>b>c. Pearson’s chi-square test with Bonferroni correction or Tukey’s post hoc comparisons was performed to assess for significant differences among the subgroups.
