# Supplementary figures and images for: Serum neurofilament light chain level as a predictor of cognitive stage transition
Source: Alzheimers Res Ther. 2022 Jan 7;14:6. doi: 10.1186/s13195-021-00953-x (PMC8742445; doi:10.1186/s13195-021-00953-x)

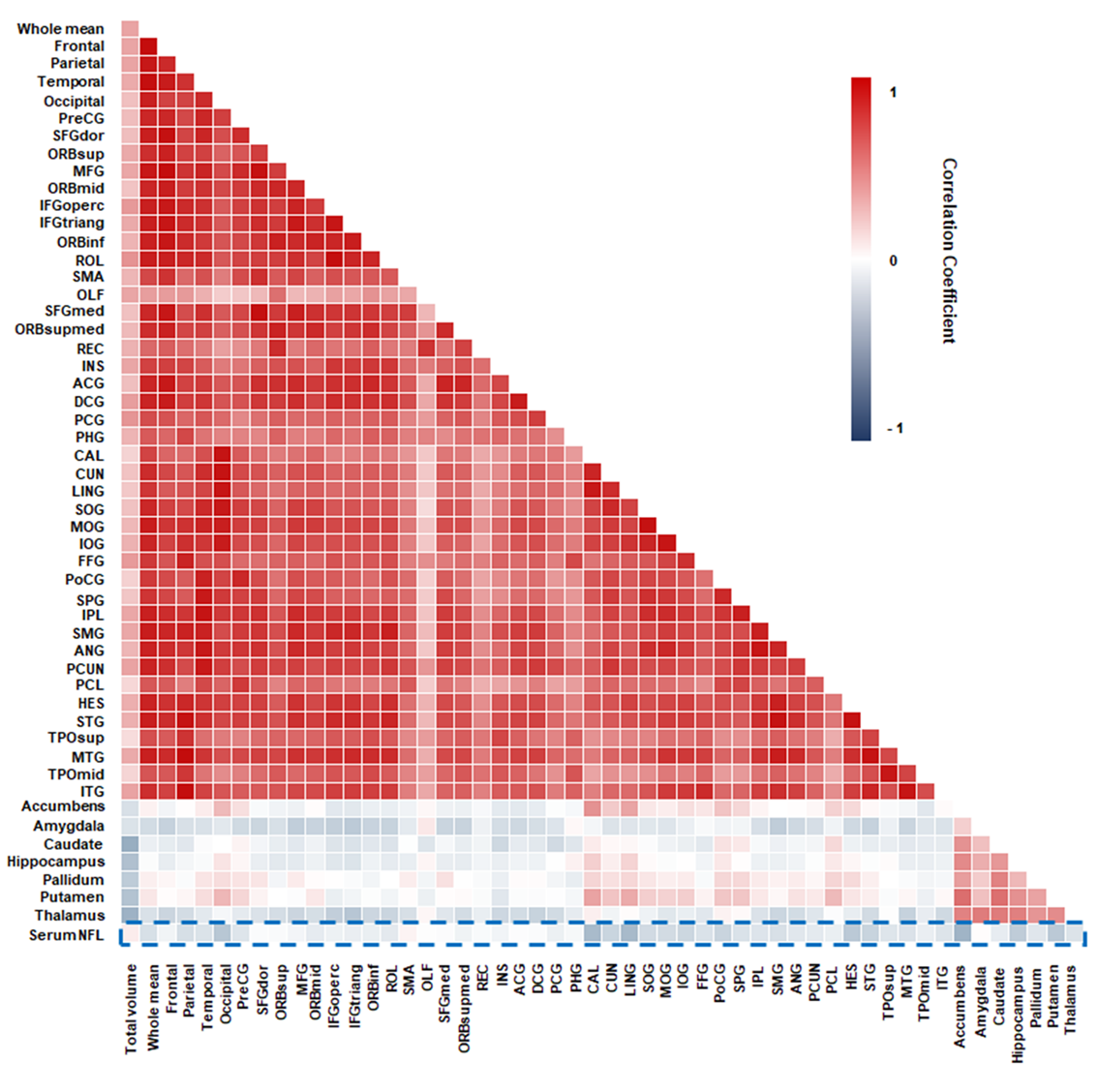

Supplement: Supplementary file 2 — Additional file 2: Supplementary Figure 1. The correlation between the cortical thickness of each specific brain region and serum NFL. The correlation between the cortical thickness of each specific brain region and serum NFL is represented in different colors. The positive and negative correlation coefficients are shown in red and blue, respectively. The cortical thickness between each brain region showed a positive correlation. NFL levels were negatively correlated with the cortical thickness of the whole-brain and specific brain regions. PreCG, precentral gyrus; SFGdor, superior frontal gyrus (dorsal); ORBsup superior orbital gyrus; MFG, middle frontal gyrus; ORBmid, middle orbital gyrus; IFGoperc, inferior frontal gyrus pars opercularis; IFGtriang, inferior frontal gyrus pars triangularis; ORBinf, inferior orbital gyrus; ROL, rolandic operculum; SMA, supplementary motor area; OLF, olfactory cortex; SFGmed, superior frontal gyrus (medial); ORBsupmed, superior frontal gyrus (medial orbital); REC, gyrus rectus; INS, insula; ACG, anterior cingulate gyrus; DCG, dorsal cingulate gyrus; PCG, posterior cingulate gyrus, PHG, parahippocampal gyrus; CAL, calcarine fissure and surrounding cortex; CUN, cuneus; LING, lingual gyrus; SOG, superior occipital gyrus; MOG, middle occipital gyrus; IOG, inferior occipital gyrus; FFG, fusiform gyrus; PoCG, postcentral gyrus; SPG, superior parietal gyrus; IPL, inferior parietal lobule; SMG, supra marginal gyrus; ANG, angular gyrus; PCUN, precuneus, PCL, paracentral lobule; HES, Heschl’s gyrus; STG, superior temporal gyrus; TPOsup, superior temporal pole; MTG, middle temporal gyrus; TPOmid, middle temporal pole; ITG, inferior temporal gyrus. [file 13195_2021_953_MOESM2_ESM.tif]

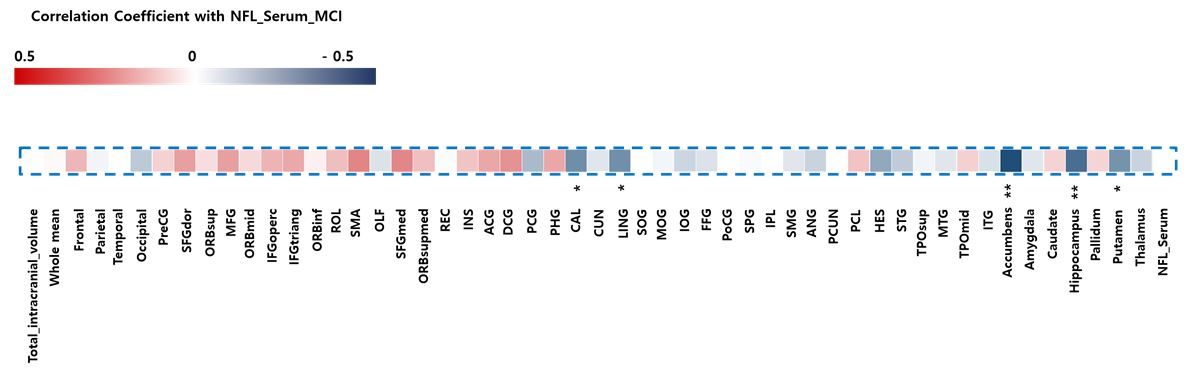

Supplement: Supplementary file 3 — Additional file 3: Supplementary Figure 2. The correlation between the cortical thickness of each specific brain region and serum NFL in mild cognitive impairment participants (n = 51). The correlation between the cortical thickness of each specific brain region and serum NFL is represented in different colors. Positive and negative correlation coefficients are shown in red and blue, respectively. NFL levels were negatively correlated with the cortical thickness of the specific brain regions including the CAL, LING, accumbens, hippocampus, and putamen. PreCG, precentral gyrus; SFGdor, superior frontal gyrus (dorsal); ORBsup superior orbital gyrus; MFG, middle frontal gyrus; ORBmid, middle orbital gyrus; IFGoperc, inferior frontal gyrus pars opercularis; IFGtriang, inferior frontal gyrus pars triangularis; ORBinf, inferior orbital gyrus; ROL, rolandic operculum; SMA, supplementary motor area; OLF, olfactory cortex; SFGmed, superior frontal gyrus (medial); ORBsupmed, superior frontal gyrus (medial orbital); REC, gyrus rectus; INS, insula; ACG, anterior cingulate gyrus; DCG, dorsal cingulate gyrus; PCG, posterior cingulate gyrus, PHG, parahippocampal gyrus; CAL, calcarine fissure and surrounding cortex; CUN, cuneus; LING, lingual gyrus; SOG, superior occipital gyrus; MOG, middle occipital gyrus; IOG, inferior occipital gyrus; FFG, fusiform gyrus; PoCG, postcentral gyrus; SPG, superior parietal gyrus; IPL, inferior parietal lobule; SMG, supra marginal gyrus; ANG, angular gyrus; PCUN, precuneus, PCL, paracentral lobule; HES, Heschl’s gyrus; STG, superior temporal gyrus; TPOsup, superior temporal pole; MTG, middle temporal gyrus; TPOmid, middle temporal pole; ITG, inferior temporal gyrus. [file 13195_2021_953_MOESM3_ESM.tif]

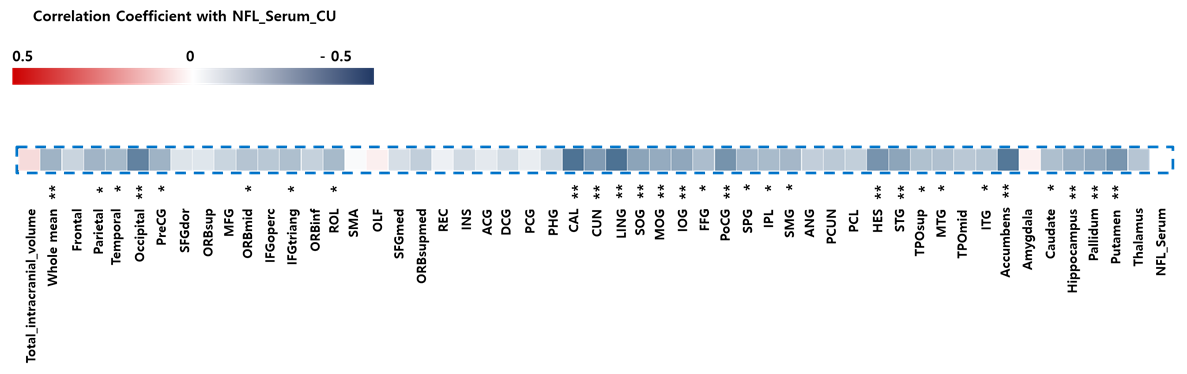

Supplement: Supplementary file 4 — Additional file 4: Supplementary Figure 3. The correlation between the cortical thickness of each specific brain region and serum NFL in cognitively unimpaired participants (n = 145). The correlation between the cortical thickness of each specific brain region and serum NFL is represented in different colors. Positive and negative correlation coefficients are shown in red and blue, respectively. NFL levels were negatively correlated with the cortical thickness of the whole-brain and specific brain regions including the parietal, temporal, and occipital cortex. PreCG, precentral gyrus; SFGdor, superior frontal gyrus (dorsal); ORBsup superior orbital gyrus; MFG, middle frontal gyrus; ORBmid, middle orbital gyrus; IFGoperc, inferior frontal gyrus pars opercularis; IFGtriang, inferior frontal gyrus pars triangularis; ORBinf, inferior orbital gyrus; ROL, rolandic operculum; SMA, supplementary motor area; OLF, olfactory cortex; SFGmed, superior frontal gyrus (medial); ORBsupmed, superior frontal gyrus (medial orbital); REC, gyrus rectus; INS, insula; ACG, anterior cingulate gyrus; DCG, dorsal cingulate gyrus; PCG, posterior cingulate gyrus, PHG, parahippocampal gyrus; CAL, calcarine fissure and surrounding cortex; CUN, cuneus; LING, lingual gyrus; SOG, superior occipital gyrus; MOG, middle occipital gyrus; IOG, inferior occipital gyrus; FFG, fusiform gyrus; PoCG, postcentral gyrus; SPG, superior parietal gyrus; IPL, inferior parietal lobule; SMG, supra marginal gyrus; ANG, angular gyrus; PCUN, precuneus, PCL, paracentral lobule; HES, Heschl’s gyrus; STG, superior temporal gyrus; TPOsup, superior temporal pole; MTG, middle temporal gyrus; TPOmid, middle temporal pole; ITG, inferior temporal gyrus. [file 13195_2021_953_MOESM4_ESM.tif]

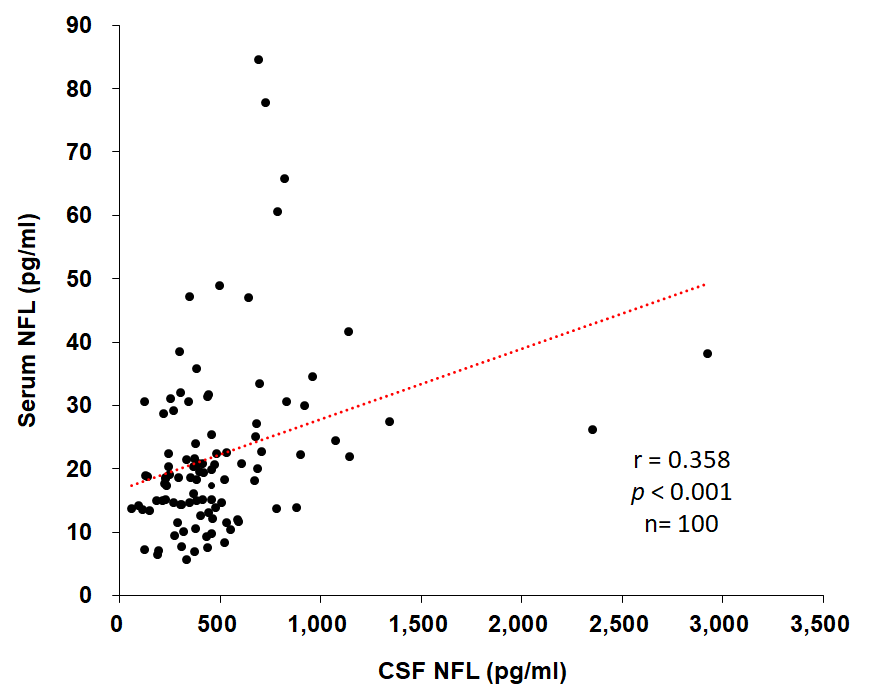

Supplement: Supplementary file 5 — Additional file 5: Supplementary Figure 4. Serum and cerebrospinal fluid neurofilament light chain according to amyloid-beta positivity. Serum and cerebrospinal fluid neurofilament light chain according to amyloid-beta positivity. Fit lines are shown for each group. The Spearman’s ρ and P values relate to Spearman’s rank correlation for each group. NFL, neurofilament light chain; CSF, cerebrospinal fluid. [file 13195_2021_953_MOESM5_ESM.tif]
